# Supplementary material for: (5-Hydroxy-4-oxo-2-styryl-4H-pyridin-1-yl)-acetic Acid Derivatives as Multifunctional Aldose Reductase Inhibitors
Source: Molecules. 2020 Nov 4;25(21):5135. doi: 10.3390/molecules25215135 (PMC7663616; doi:10.3390/molecules25215135)
Supplement: Supplementary file 1 [file molecules-25-05135-s001.zip › molecules-972985-supplementary/molecules-972985-supplementary - Copy.pdf]

molecules

MDPI

Supporting Information

(5-Hydroxy-4-oxo-2-styryl-4H-pyridin-1-yl)-acetic Acid Derivatives as Multifunctional Aldose Reductase Inhibitors

Huan Chen, Xin Zhang, Xiaonan Zhang, Wenchao Liu, Yanqi Lei, Changjin Zhu * and Bing Ma *

School of Chemistry and Chemical Engineering, Beijing Institute of Technology, Beijing 100081, China; ch13264138964@163.com (H.C.); zx15811458104@163.com (X.Z.); zhangxiaonan965@163.com (X.Z.); liuwhhh@163.com (W.L.); 15313252939@163.com (Y.L.)

* Correspondence: mabing@bit.edu.cn (B.M.); zcj@bit.edu.cn (C.Z.); Tel.: +86-010-68918506 (B.M. & C.Z.)

Academic Editors: Jiang Wang, Liang-Ren Zhang, Peng Zhan, Qi-Dong You, Tian-Miao Ou and Xiao-Yun Lu

Received: 5 October 2020; Accepted: 3 November 2020; Published: 4 November 2020

NMR spectra, high-resolution mass spectra and UV-vis spectra for tested products

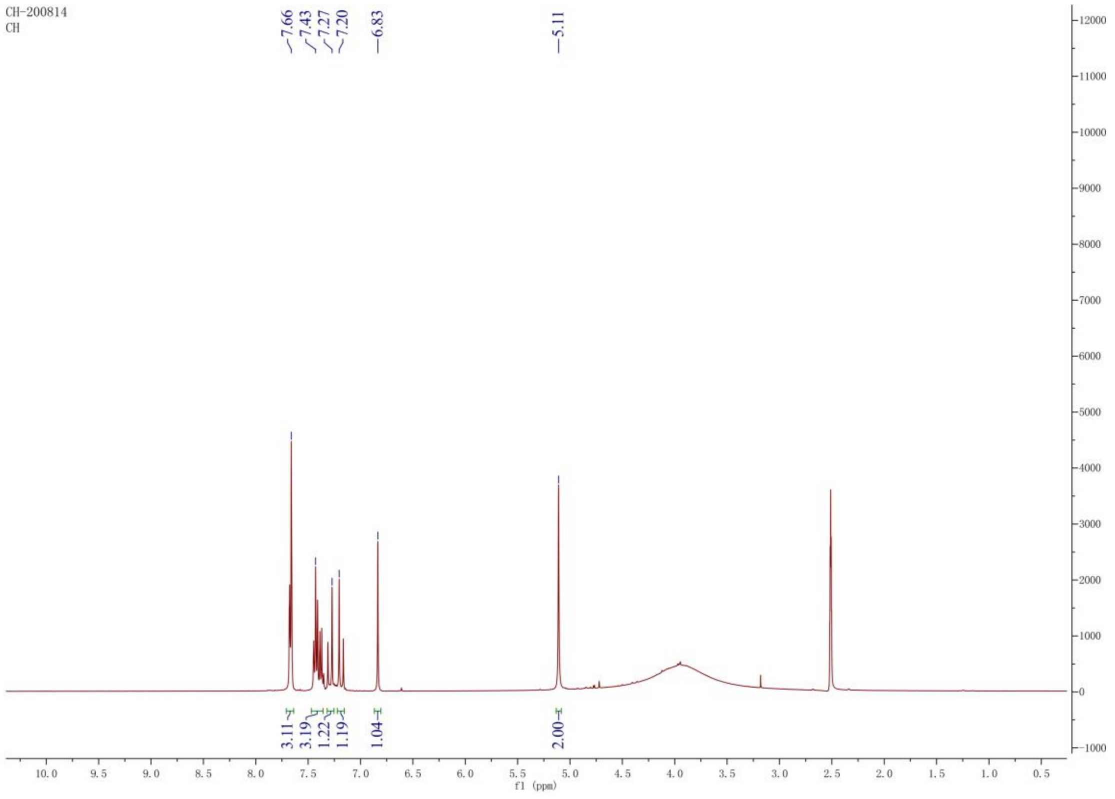

Figure S1 ^{1}H-NMR (400 MHz, DMSO-*d6*) of **7a**

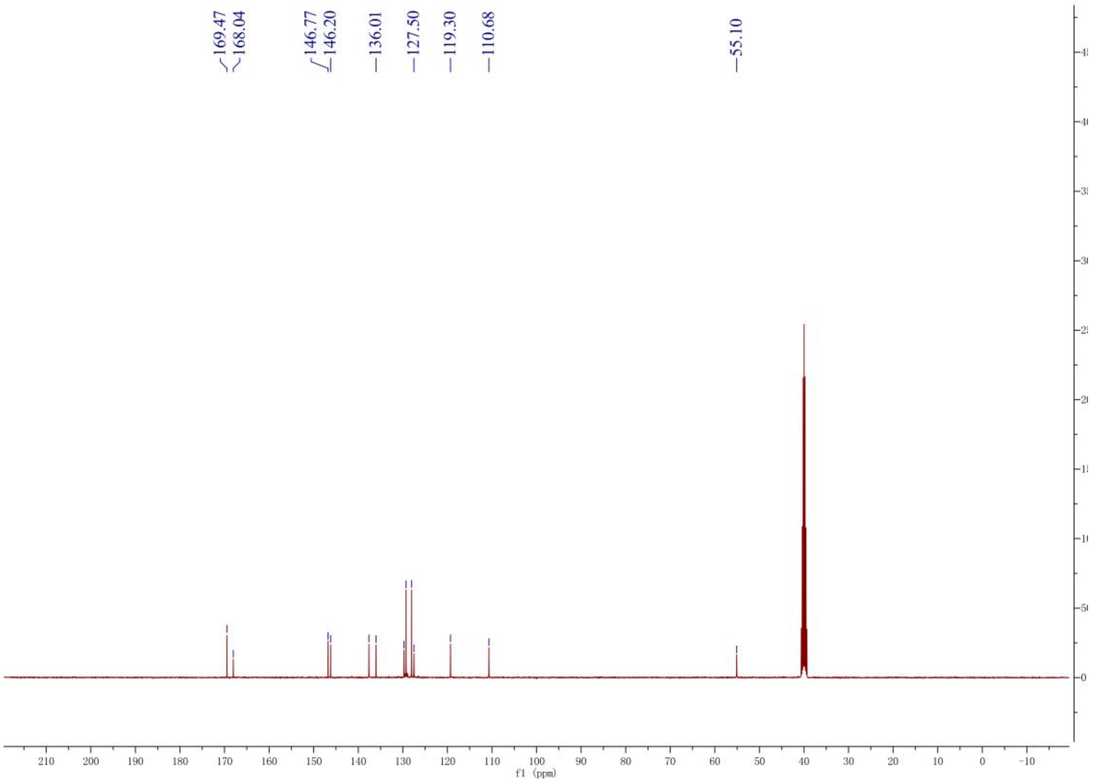

Figure S2 ^{13}C-NMR (100 MHz, DMSO-*d6*) of **7a**

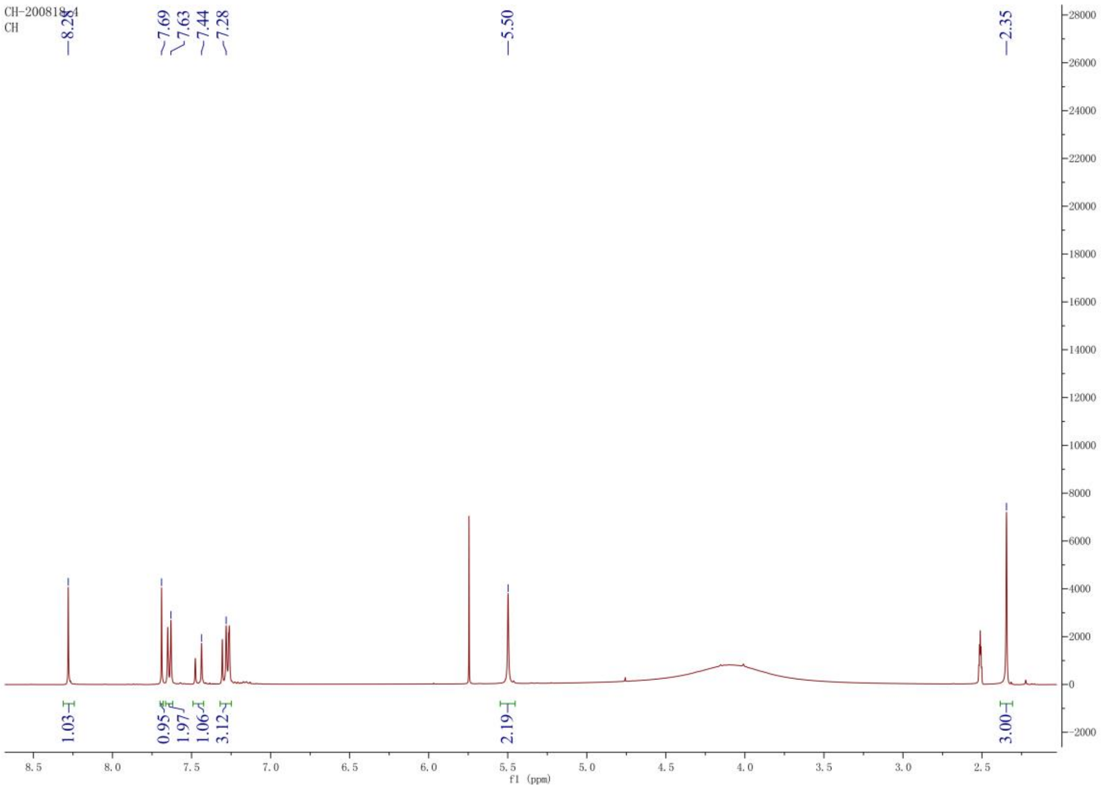

Figure S3 ^{1}H-NMR (400 MHz, DMSO-*d6*) of **7b**

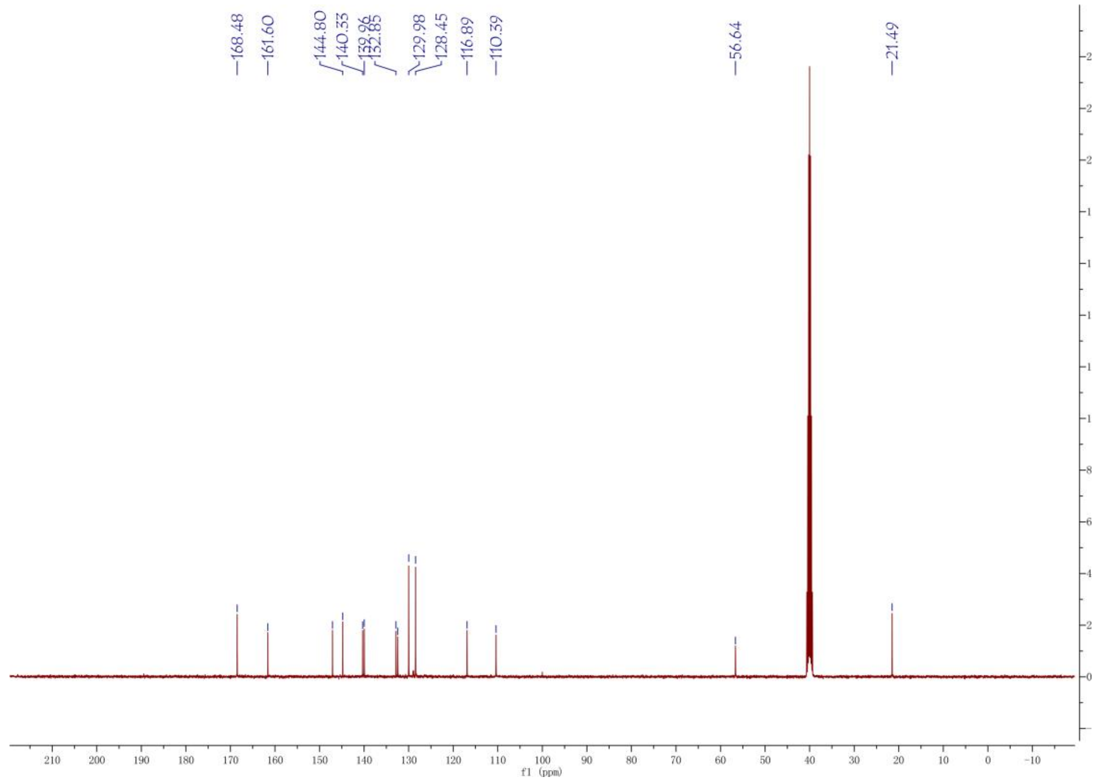

Figure S4 ^{13}C-NMR (100 MHz, DMSO-*d6*) of **7b**

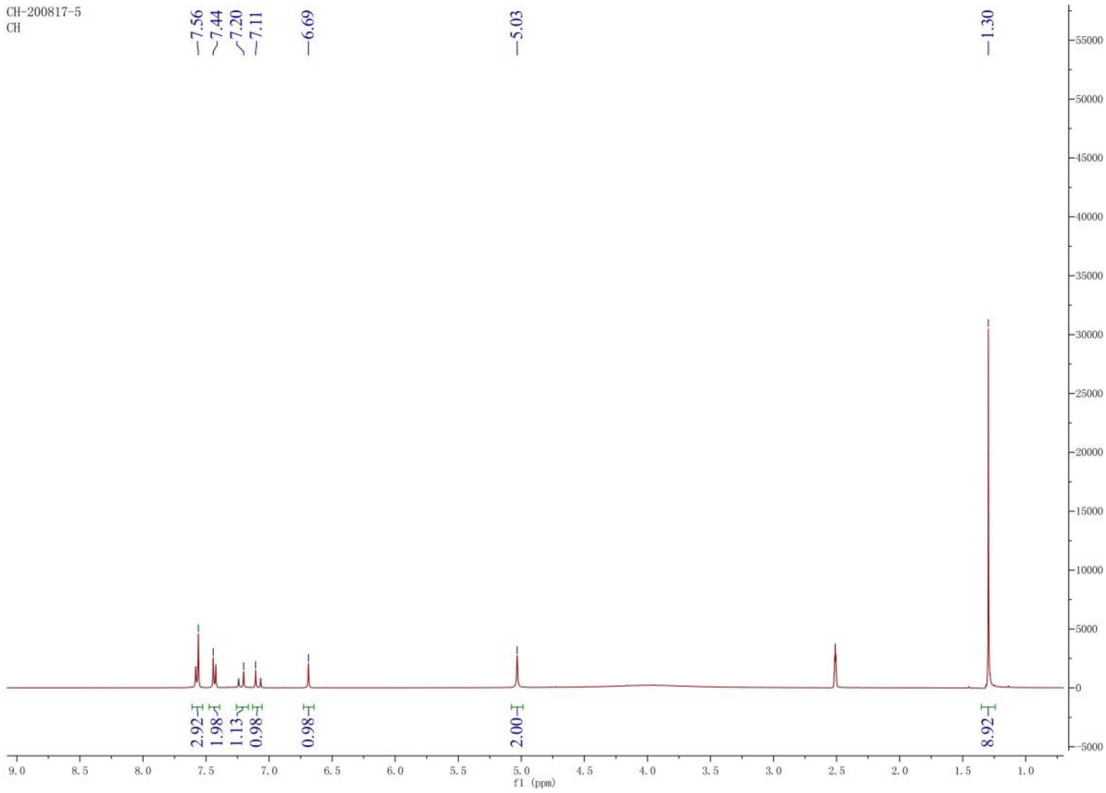

Figure S5 ^{1}H-NMR (400 MHz, DMSO-*d6*) of **7c**

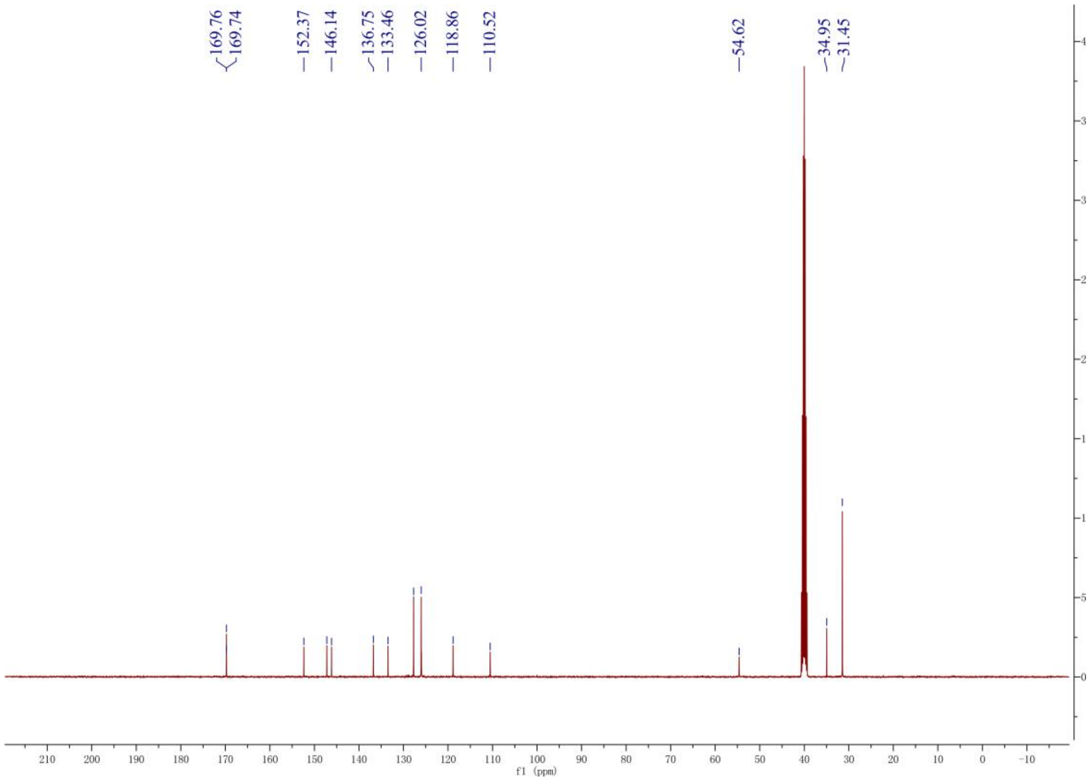

Figure S6 ^{13}C-NMR (100 MHz, DMSO-*d6*) of **7c**

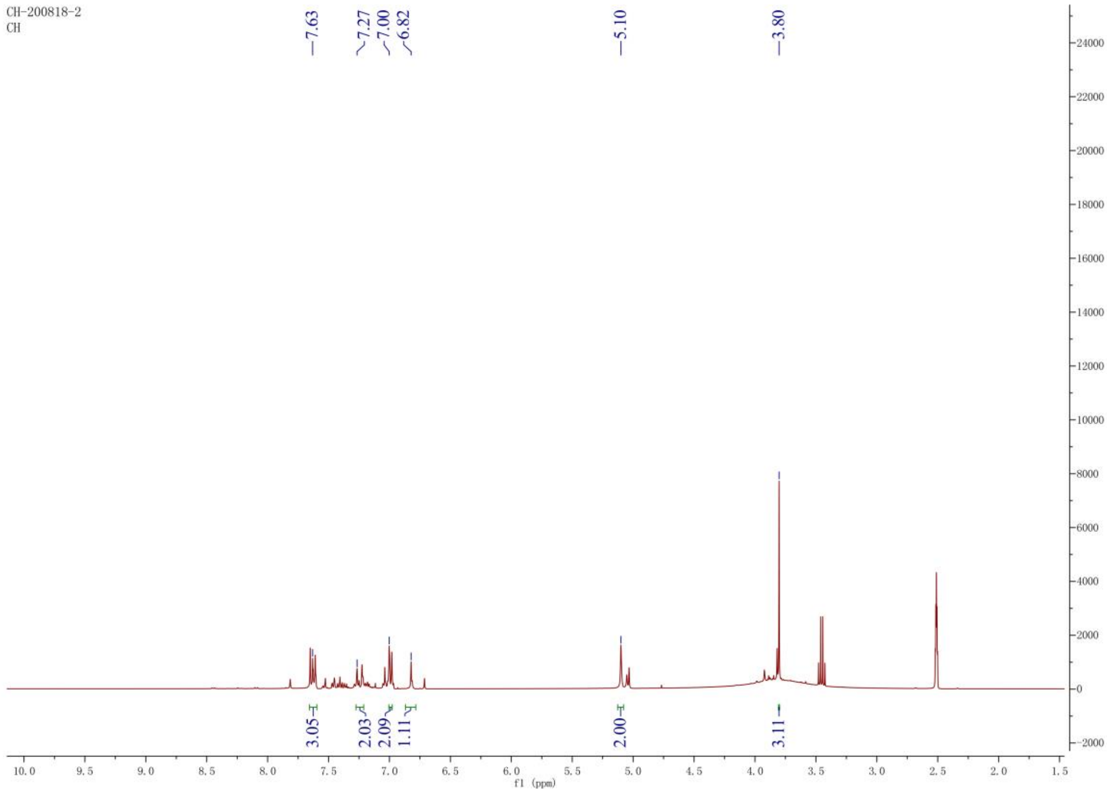

Figure S7 ^{1}H-NMR (400 MHz, DMSO-*d6*) of **7d**

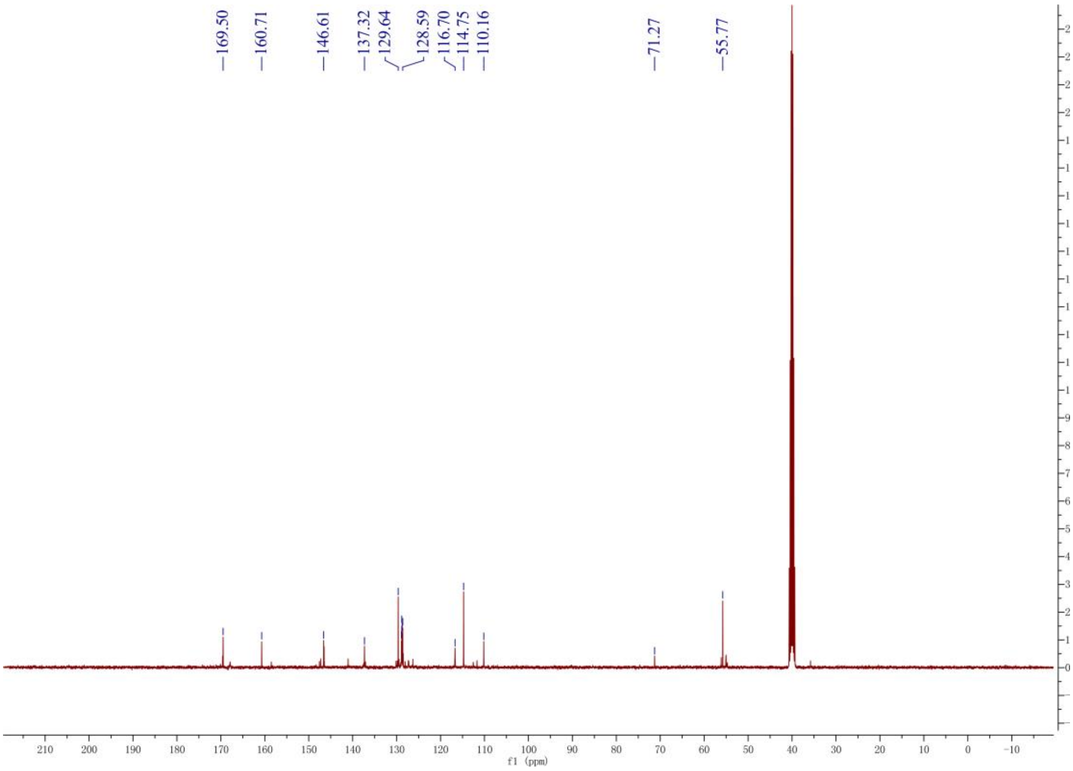

Figure S8 ^{13}C-NMR (100 MHz, DMSO-*d6*) of **7d**

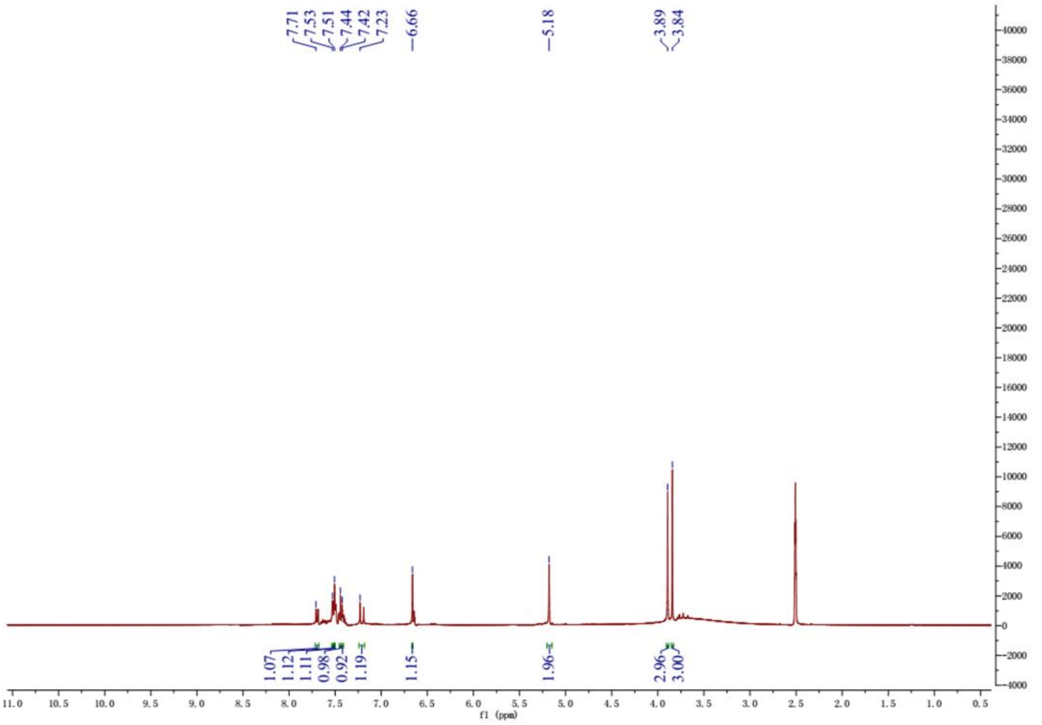

Figure S9 ^{1}H-NMR (400 MHz, DMSO-*d6*) of **7e**

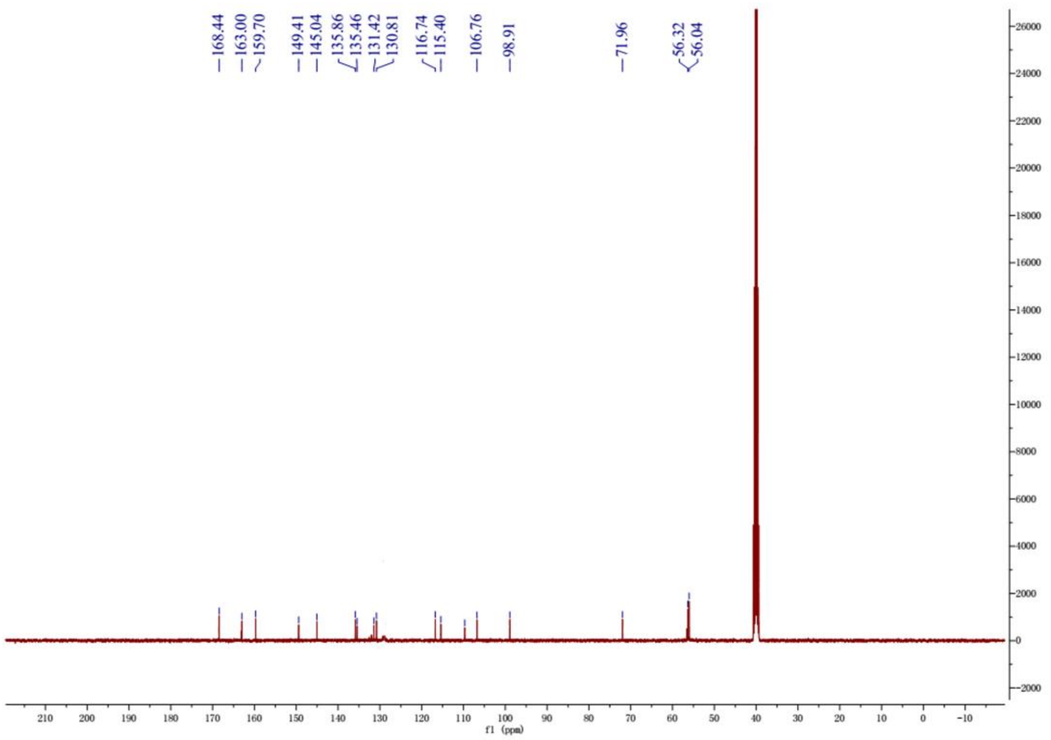

Figure S10 ^{13}C-NMR (100 MHz, DMSO-*d6*) of **7e**

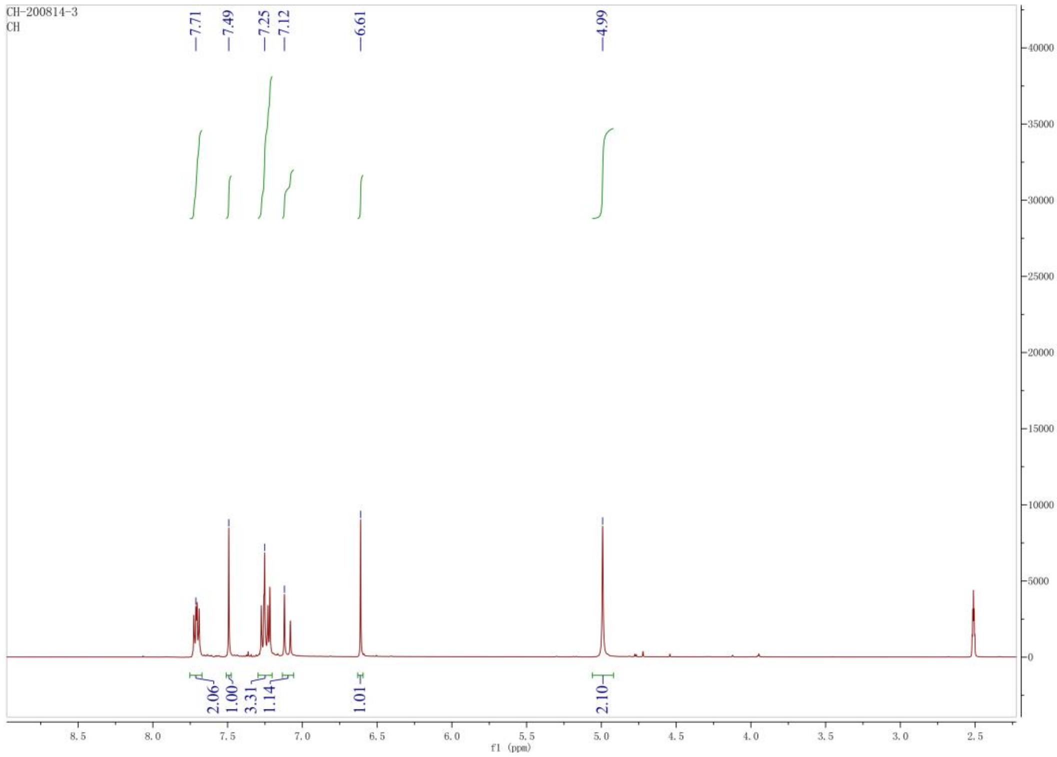

Figure S11 ^{1}H-NMR (400 MHz, DMSO-*d6*) of **7f**

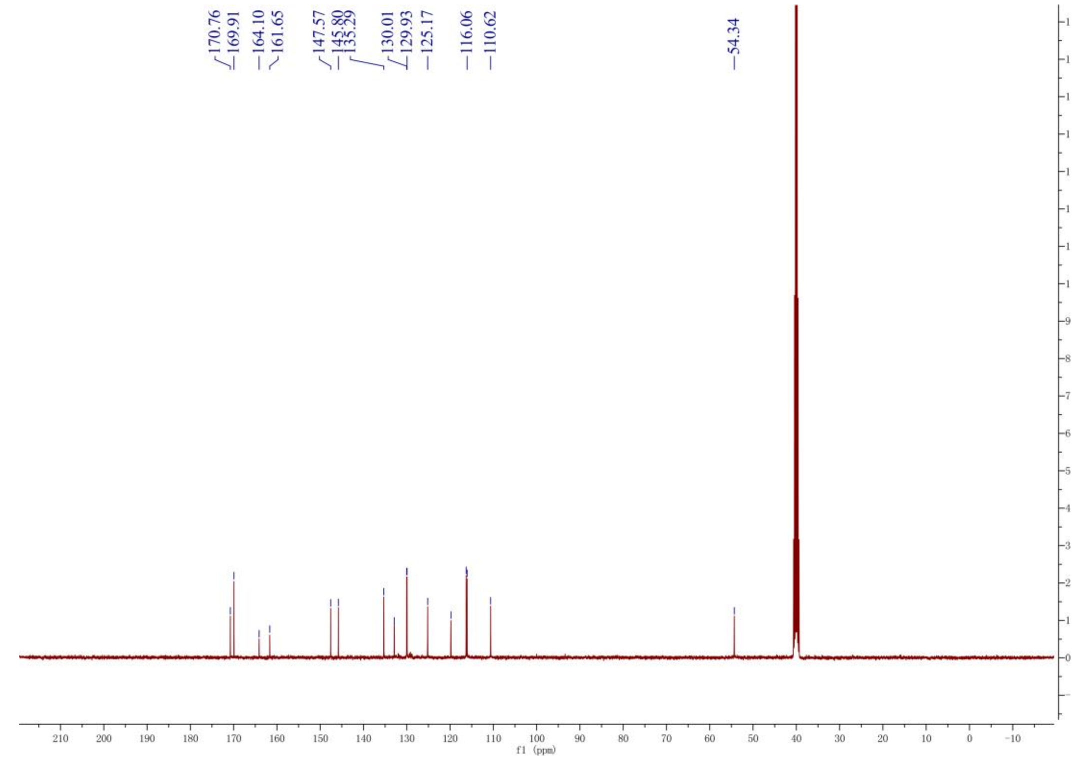

Figure S12 ^{13}C-NMR (100 MHz, DMSO-*d6*) of **7f**

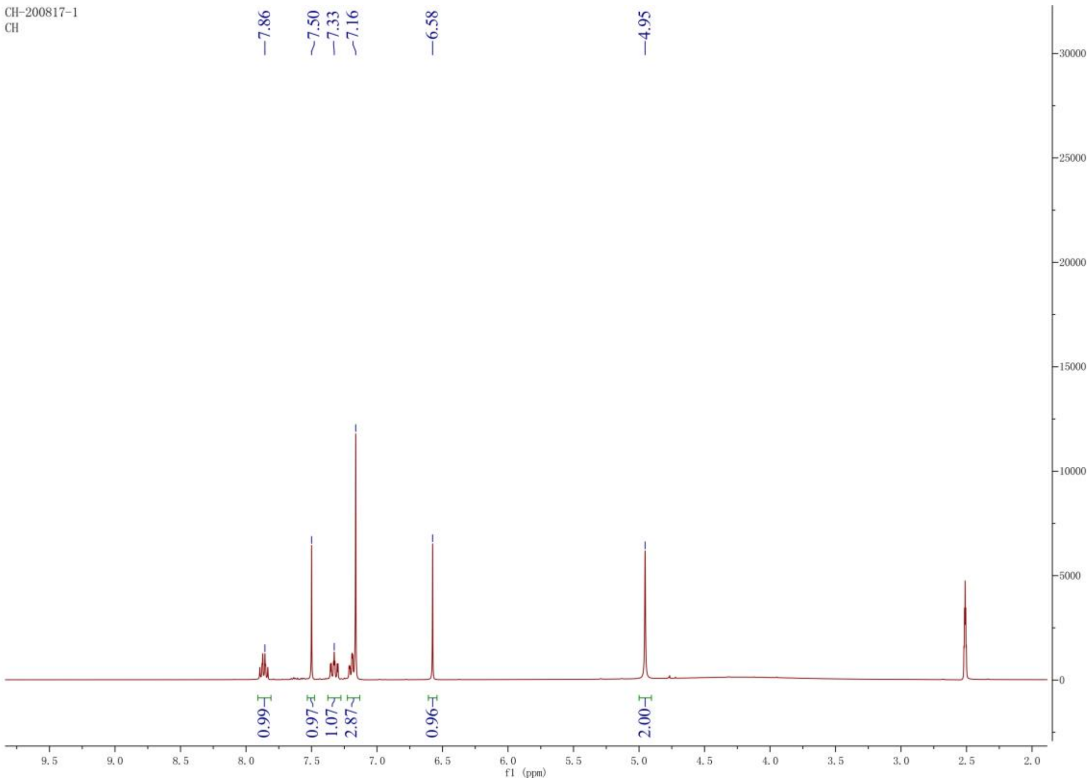

Figure S13 ^{1}H-NMR (400 MHz, DMSO-*d6*) of **7g**

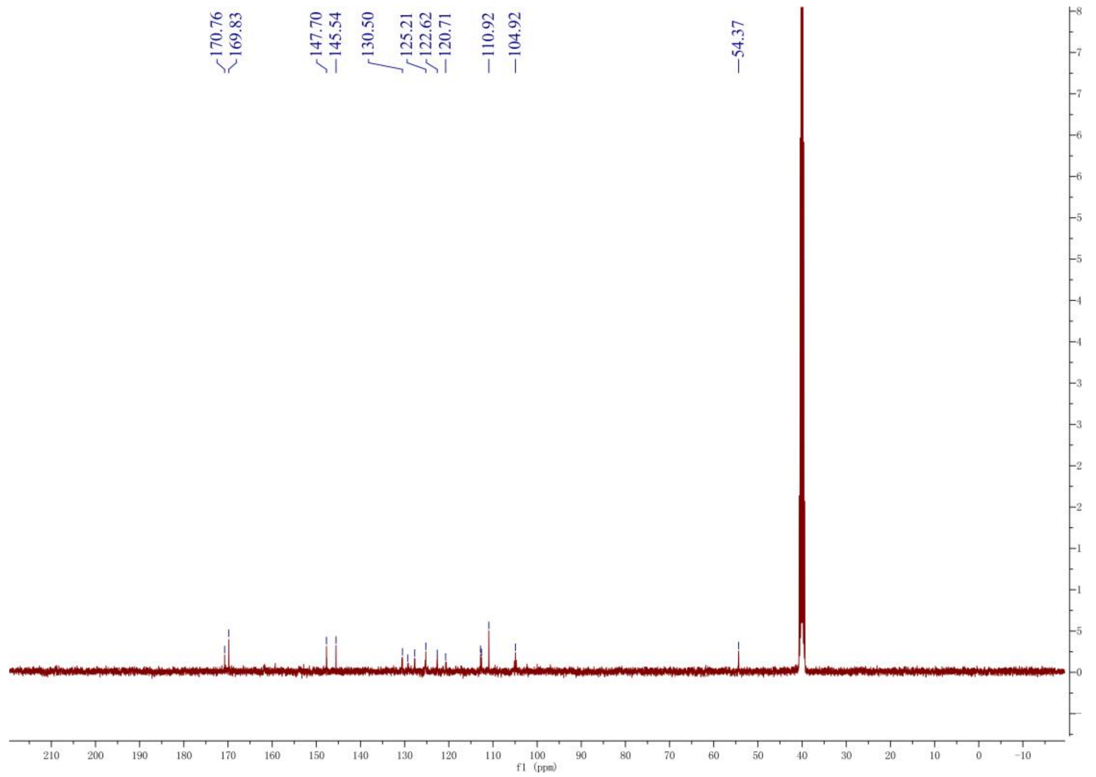

Figure S14 ^{13}C-NMR (100 MHz, DMSO-*d6*) of **7g**

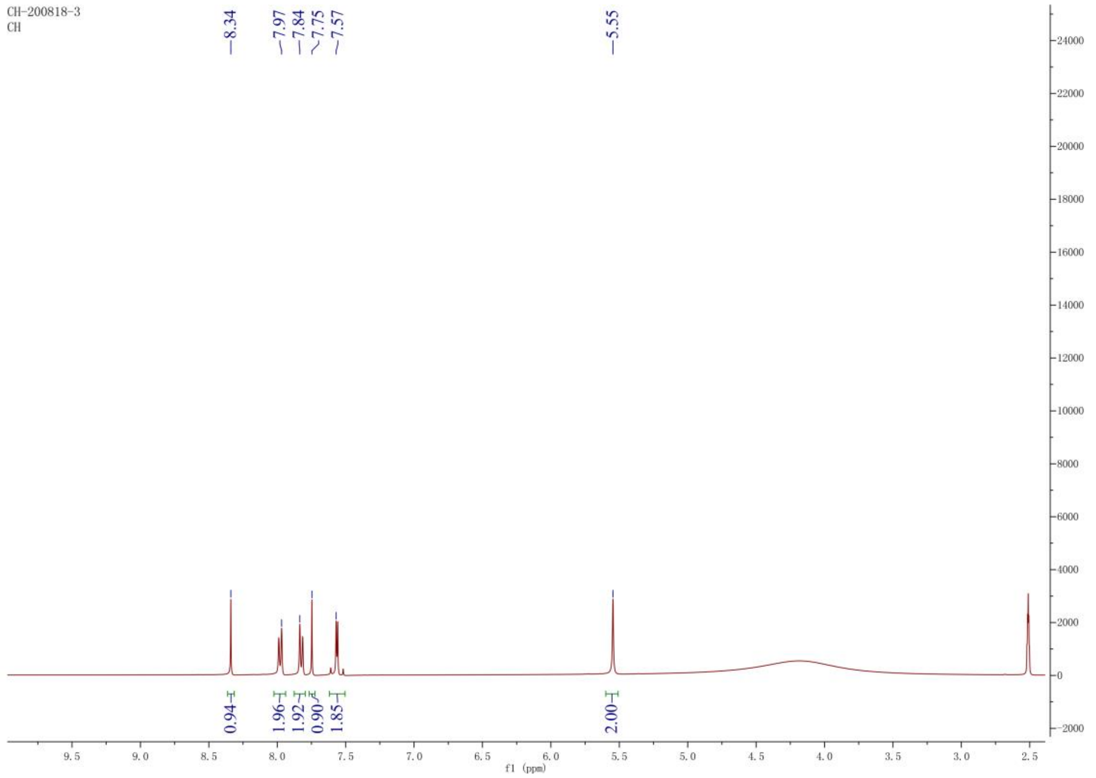

Figure S15 ^{1}H-NMR (400 MHz, DMSO-*d6*) of **7h**

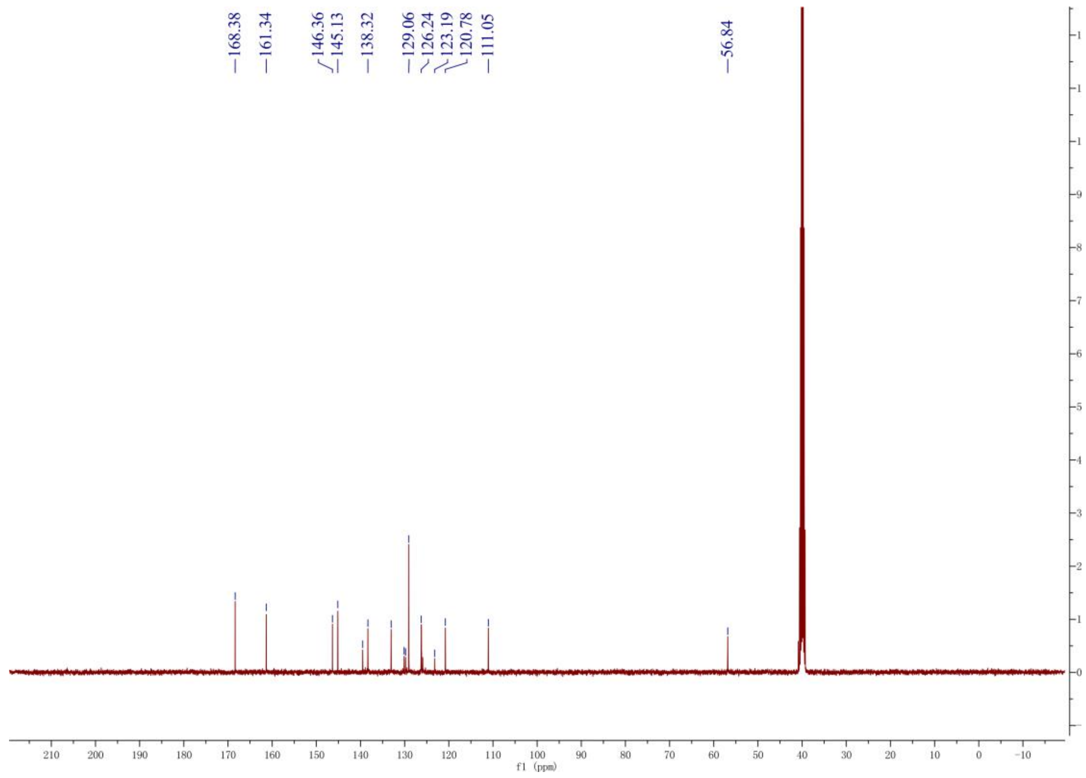

Figure S16 ^{13}C-NMR (100 MHz, DMSO-*d6*) of **7h**

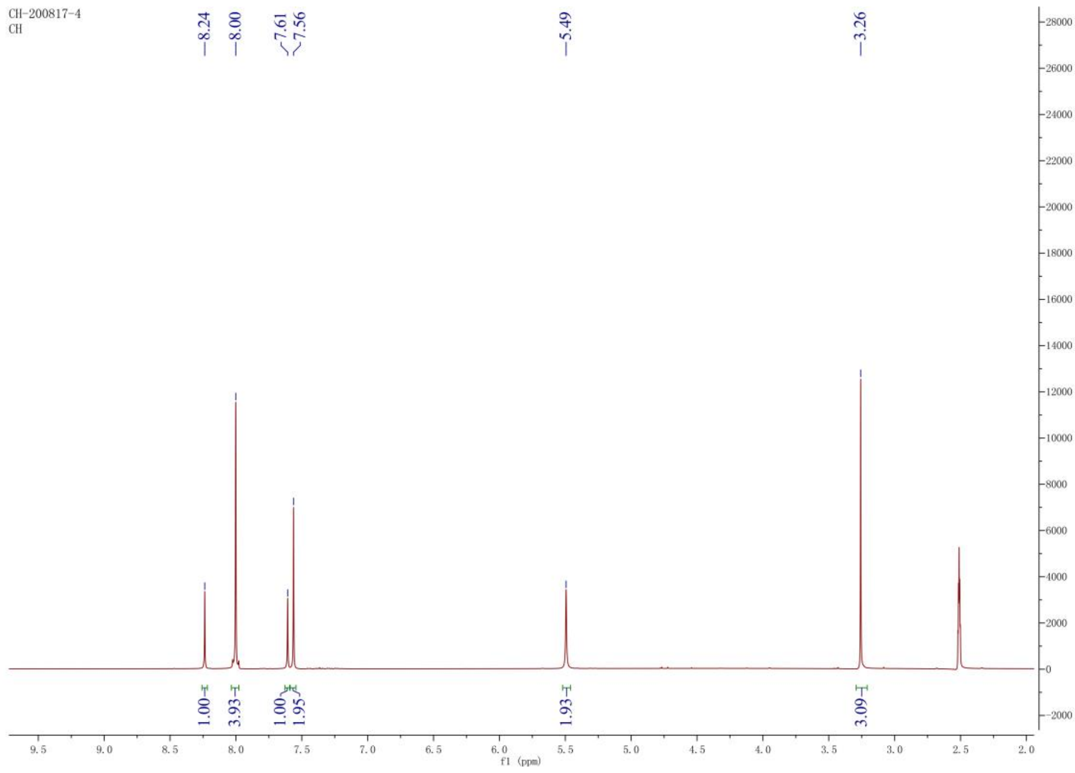

Figure S17 ^{1}H-NMR (400 MHz, DMSO-*d6*) of **7i**

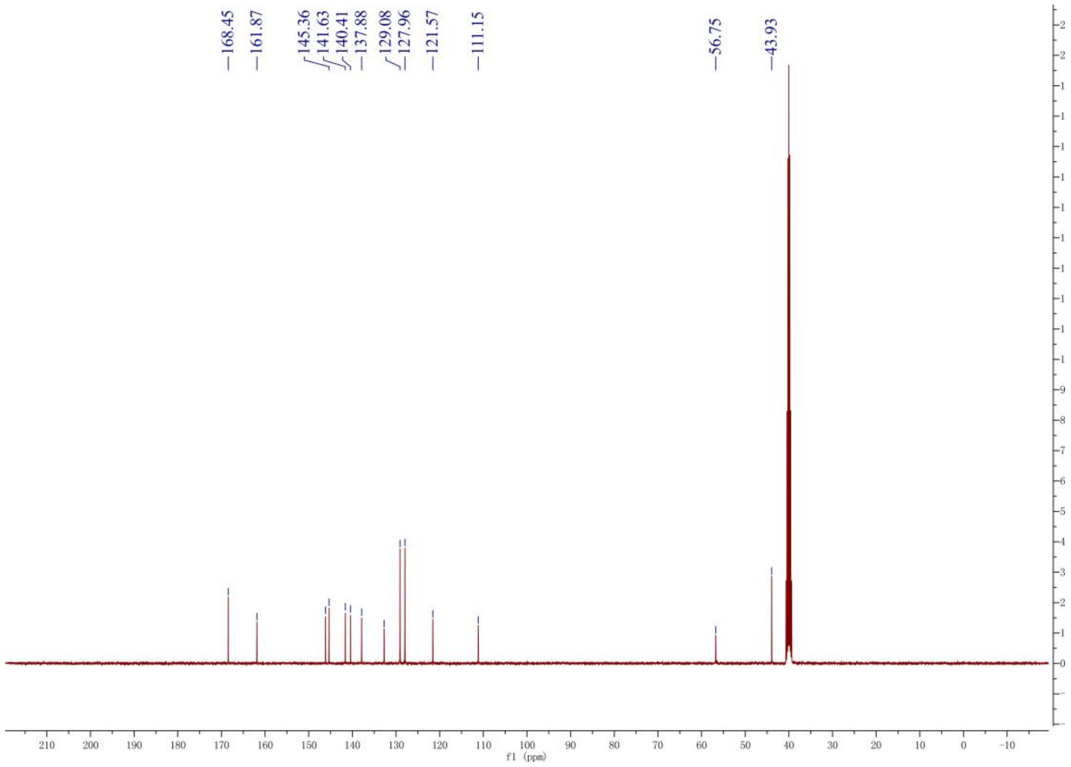

Figure S18 ^{13}C-NMR (100 MHz, DMSO-*d6*) of **7i**

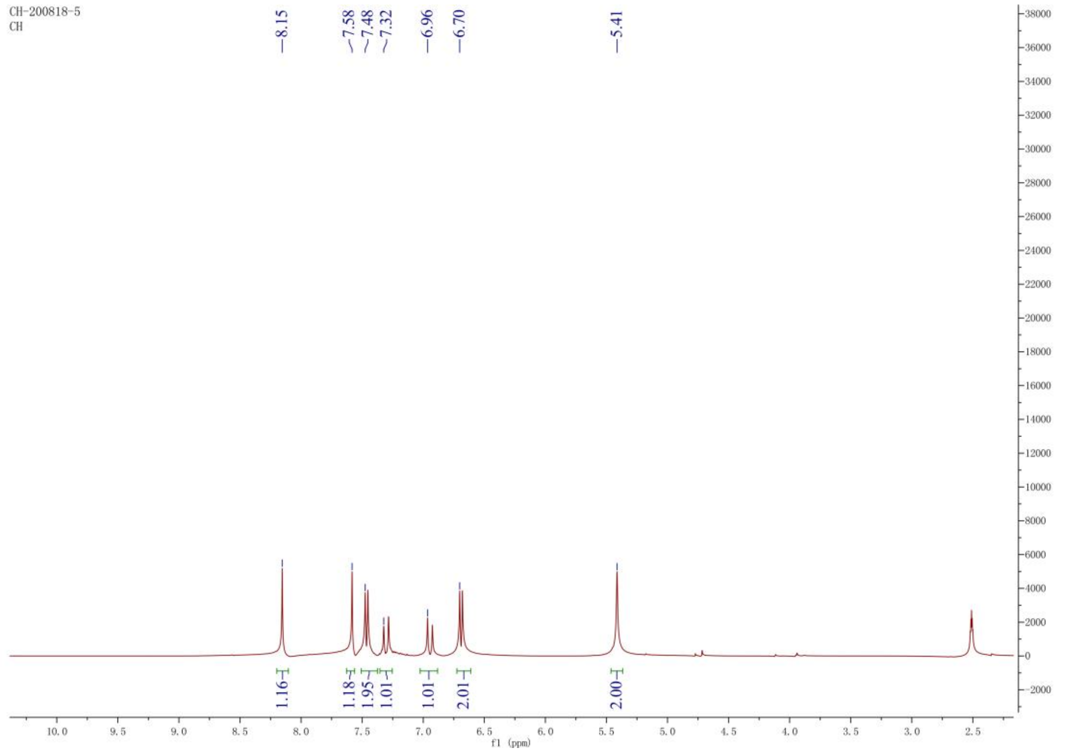

Figure S19 ^{1}H-NMR (400 MHz, DMSO-*d6*) of **7j**

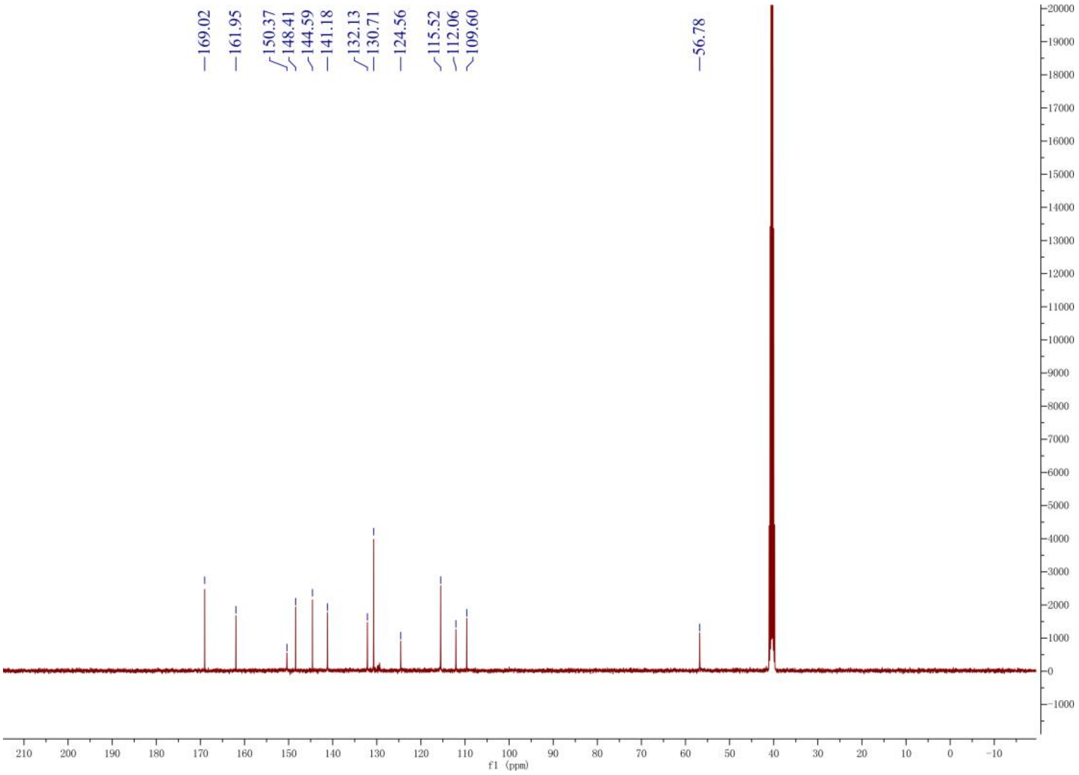

Figure S20 ^{13}C-NMR (100 MHz, DMSO-*d6*) of **7j**

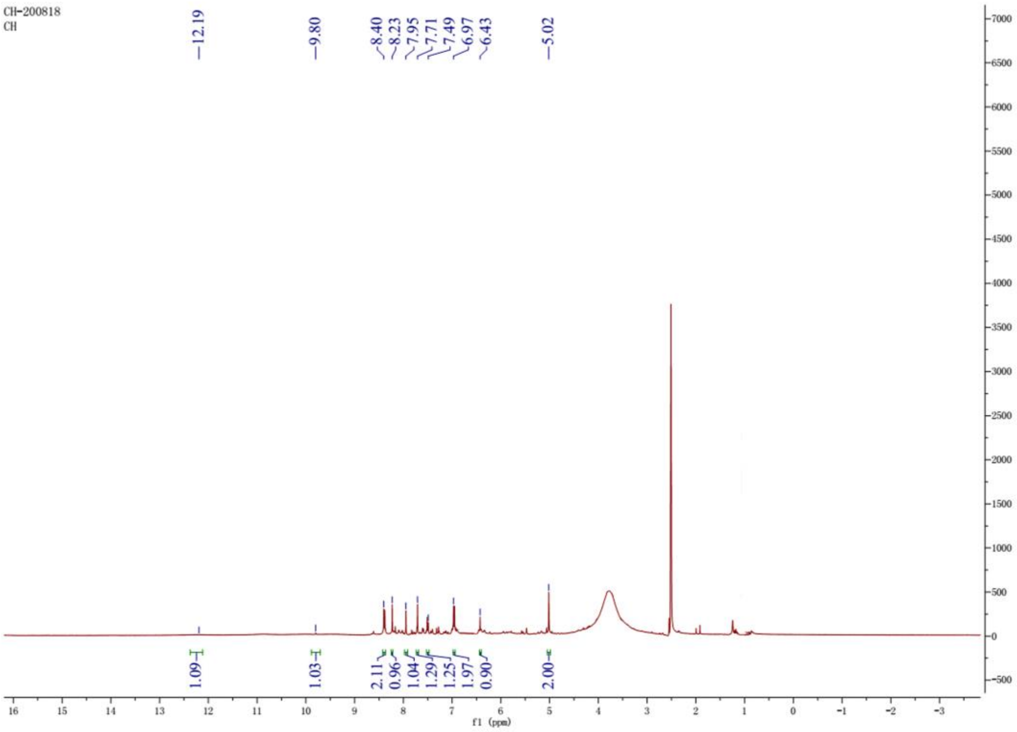

Figure S21 ^{1}H-NMR (400 MHz, DMSO-*d6*) of **7k**

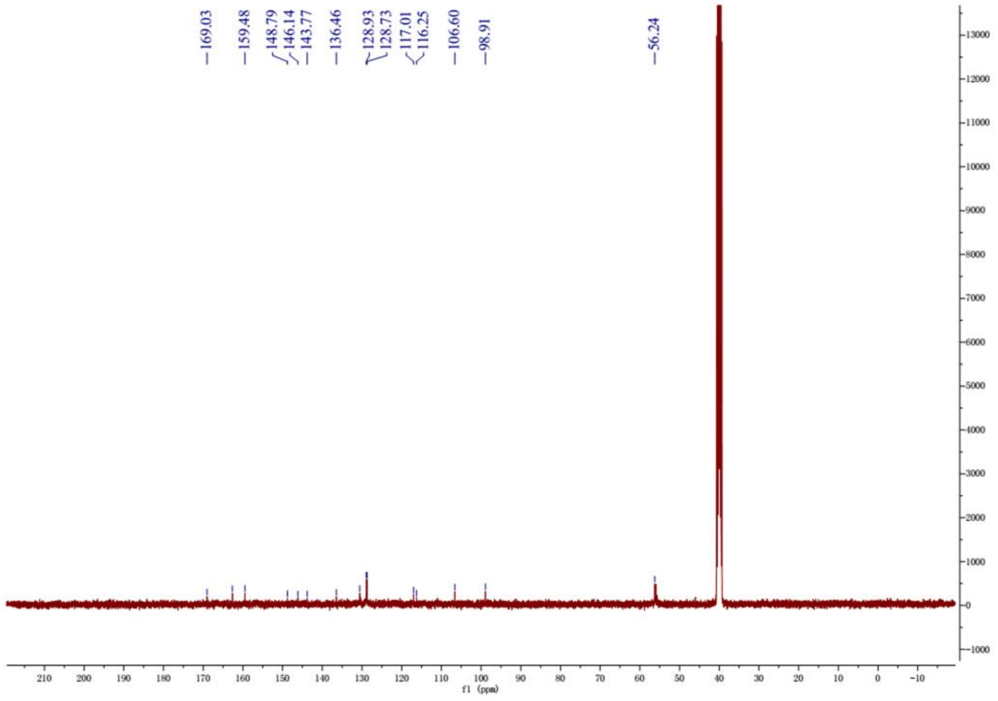

Figure S22 ^{13}C-NMR (100 MHz, DMSO-*d6*) of **7k**

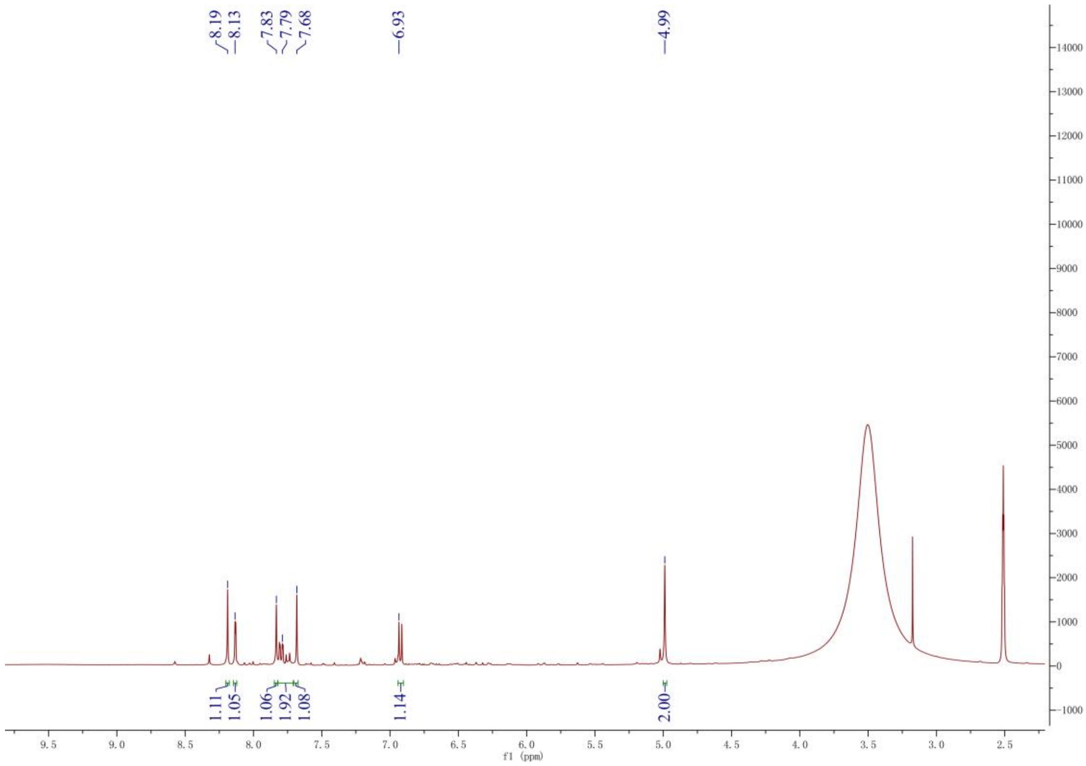

Figure S23 ^{1}H-NMR (400 MHz, DMSO-*d6*) of **7k**

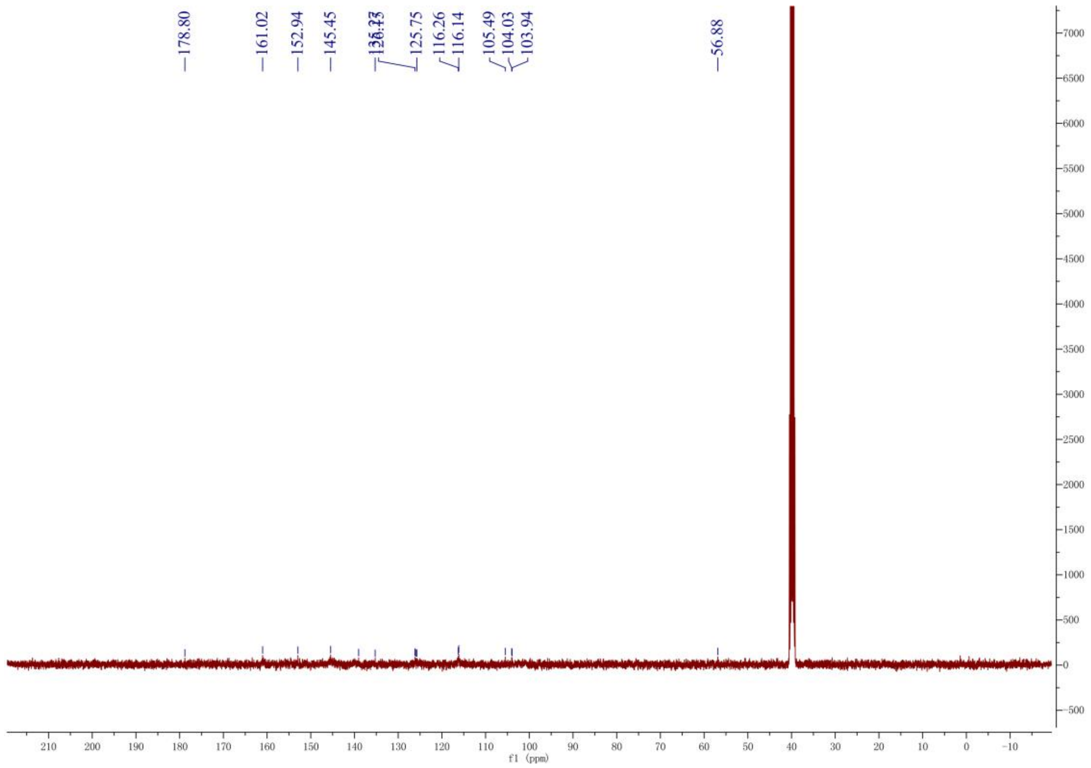

Figure S24 ^{13}C-NMR (100 MHz, DMSO-*d6*) of **7l**

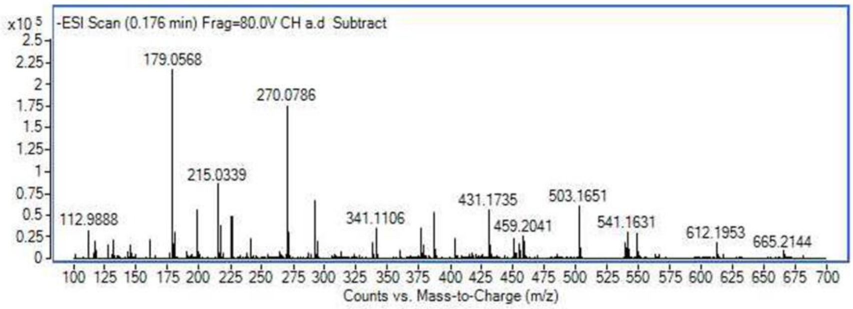

Figure S25 HRMS of **7a**

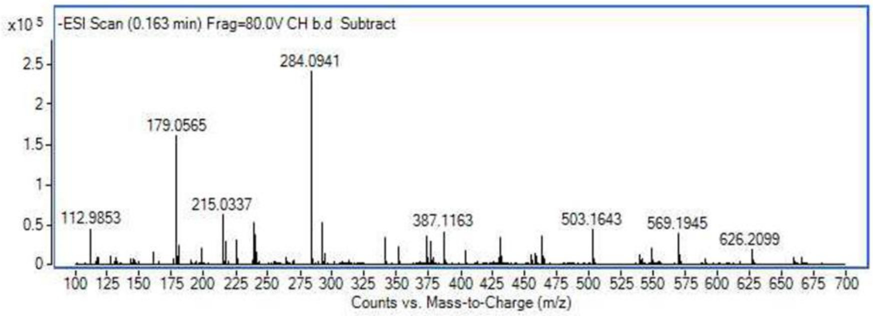

Figure S26 HRMS of **7b**

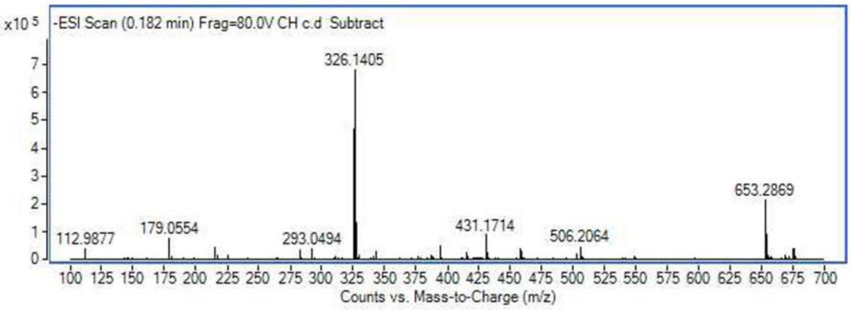

Figure S27 HRMS of **7c**

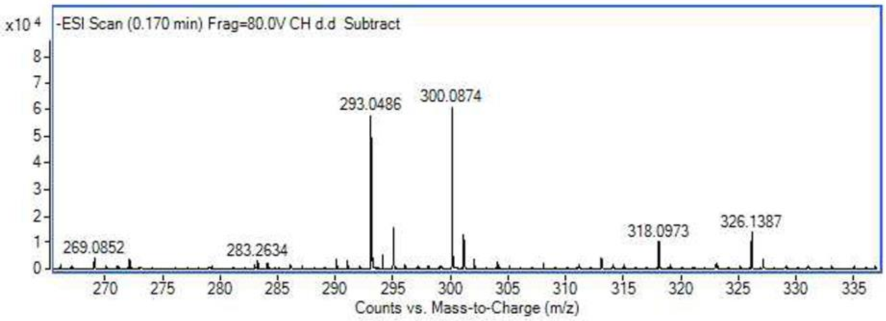

Figure S28 HRMS of **7d**

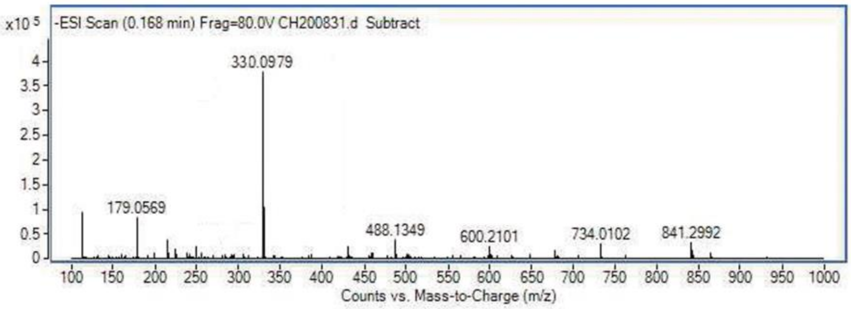

Figure S29 HRMS of **7e**

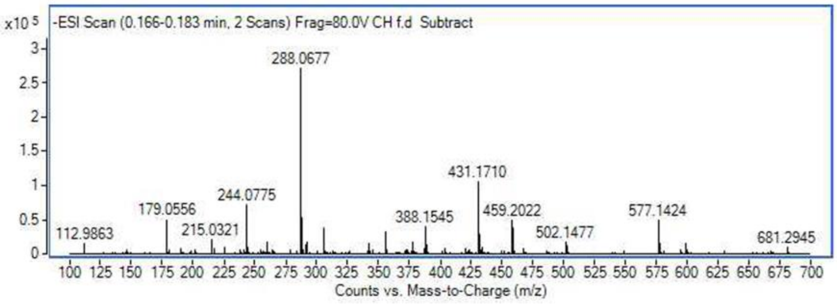

Figure S30 HRMS of **7f**

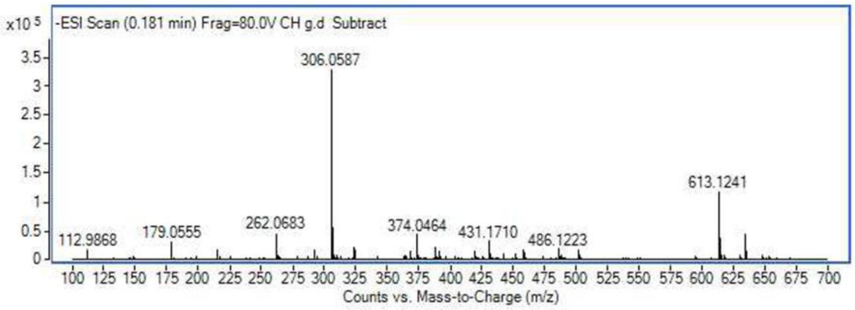

Figure S31 HRMS of **7g**

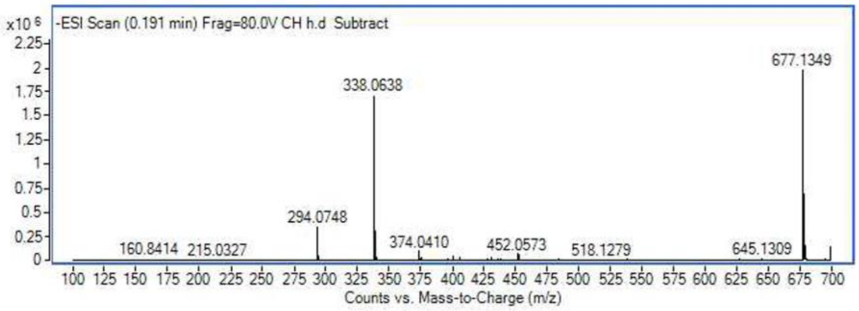

Figure S32 HRMS of **7h**

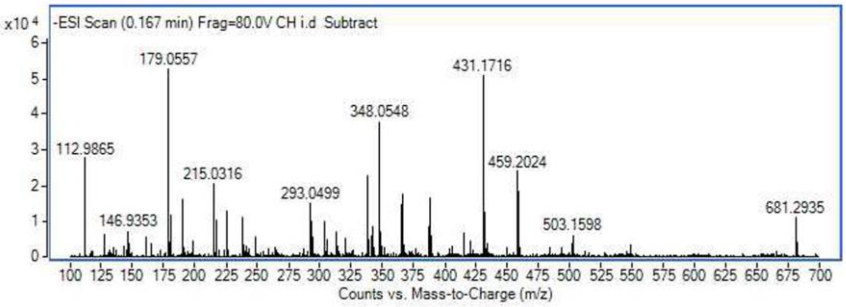

Figure S33 HRMS of **7i**

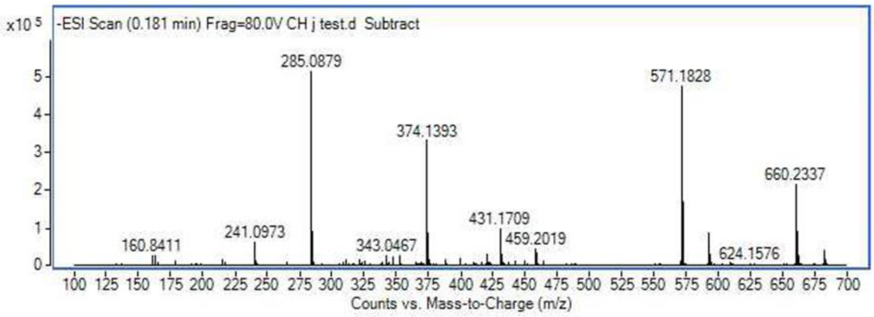

Figure S34 HRMS of **7j**

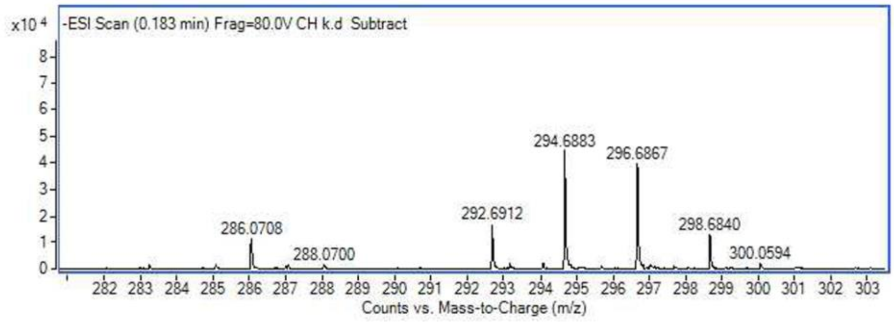

Figure S35 HRMS of **7k**

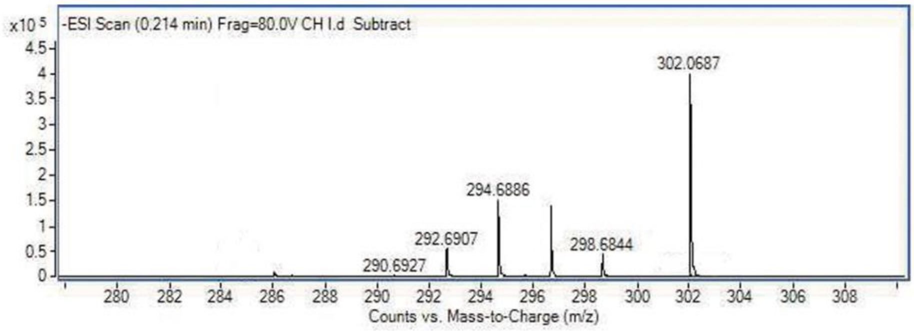

Figure S36 HRMS of **7l**

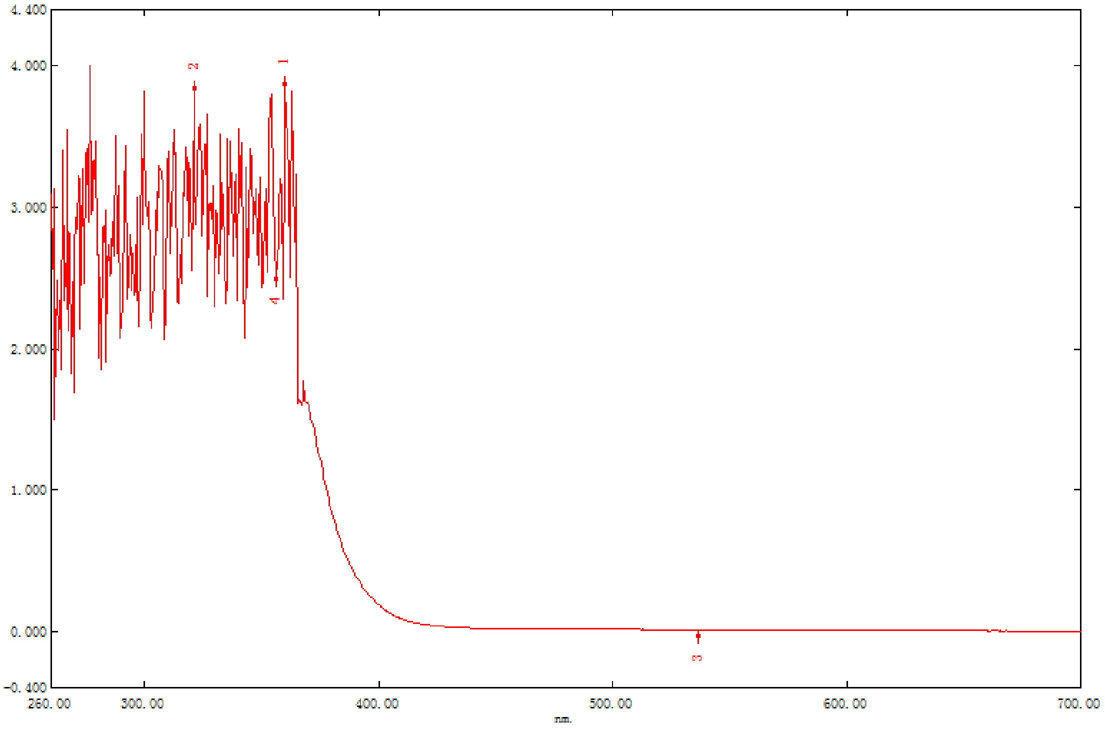

Figure S37 UV-Vis specta of **7a**

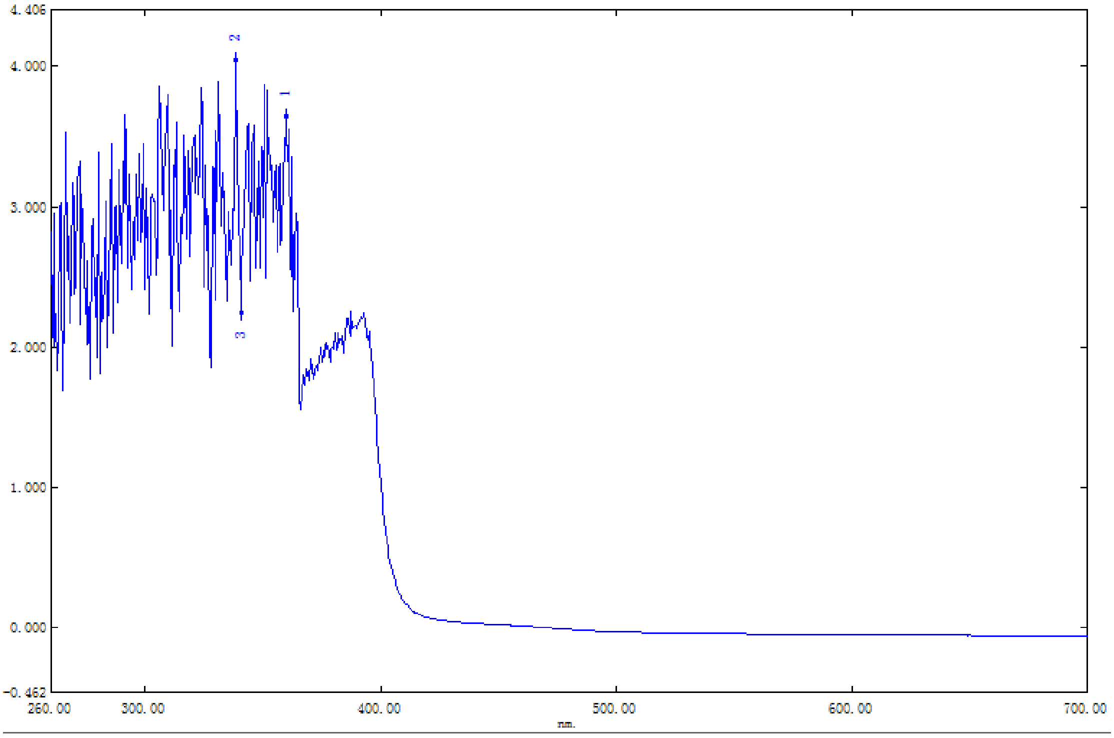

Figure S38 UV-Vis specta of **7b**

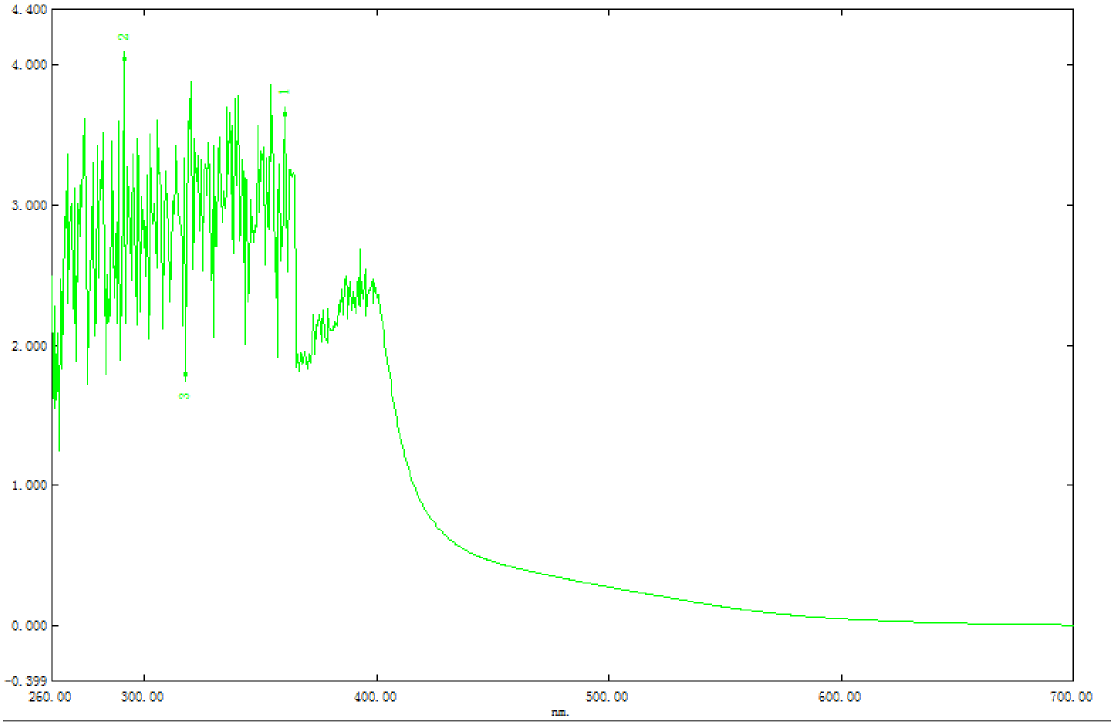

Figure S39 UV-Vis specta of **7c**

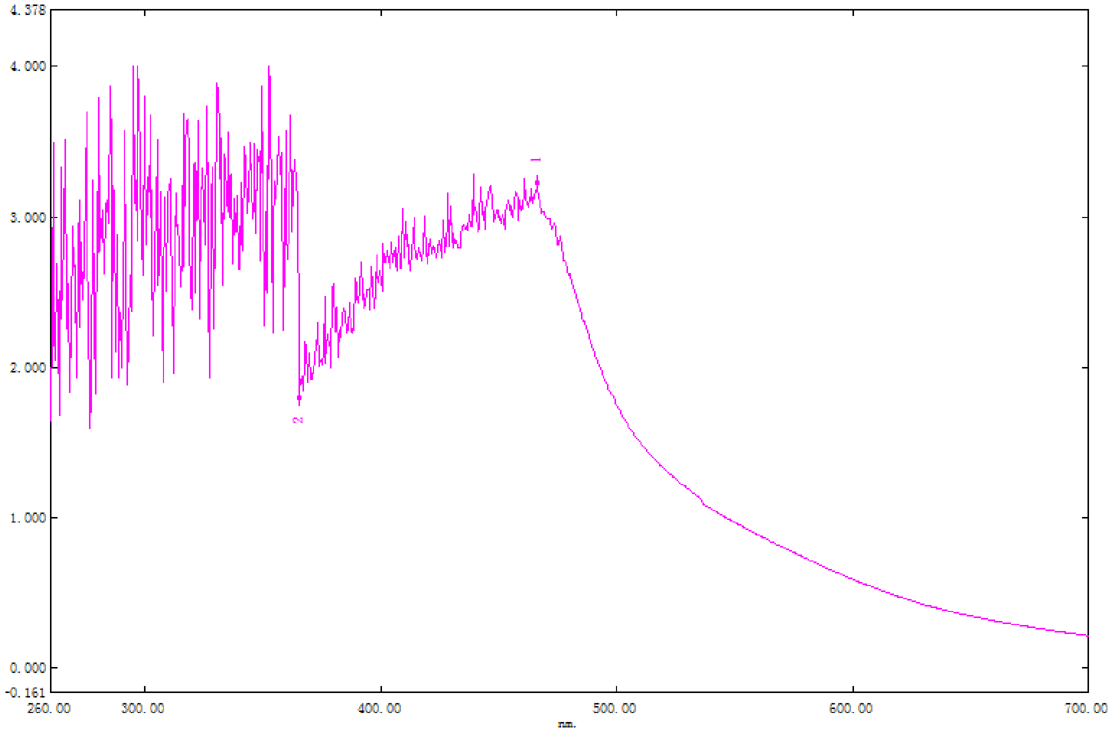

Figure S40 UV-Vis specta of **7d**

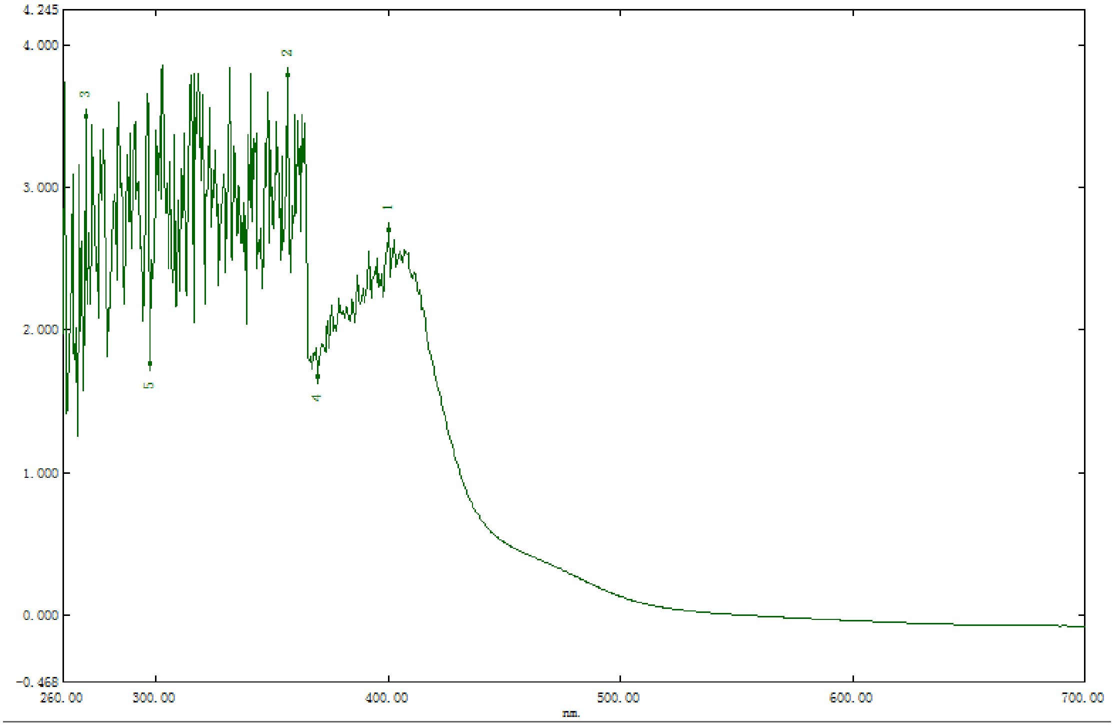

Figure S41 UV-Vis specta of **7e**

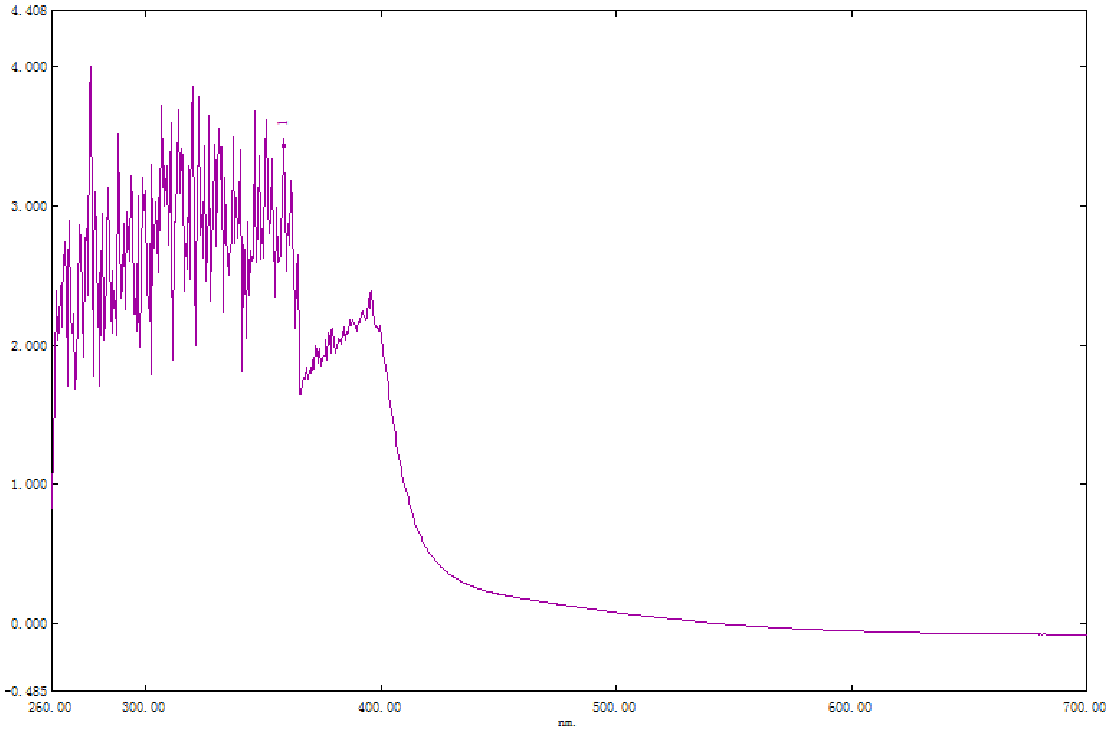

Figure S42 UV-Vis specta of **7f**

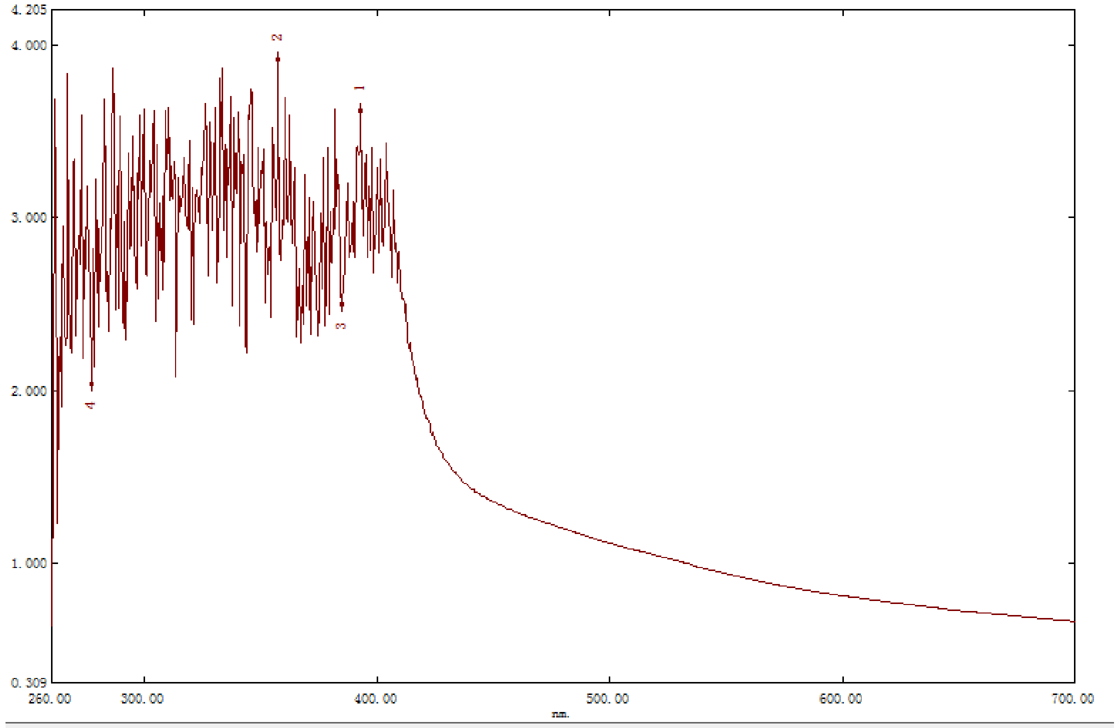

Figure S43 UV-Vis specta of **7g**

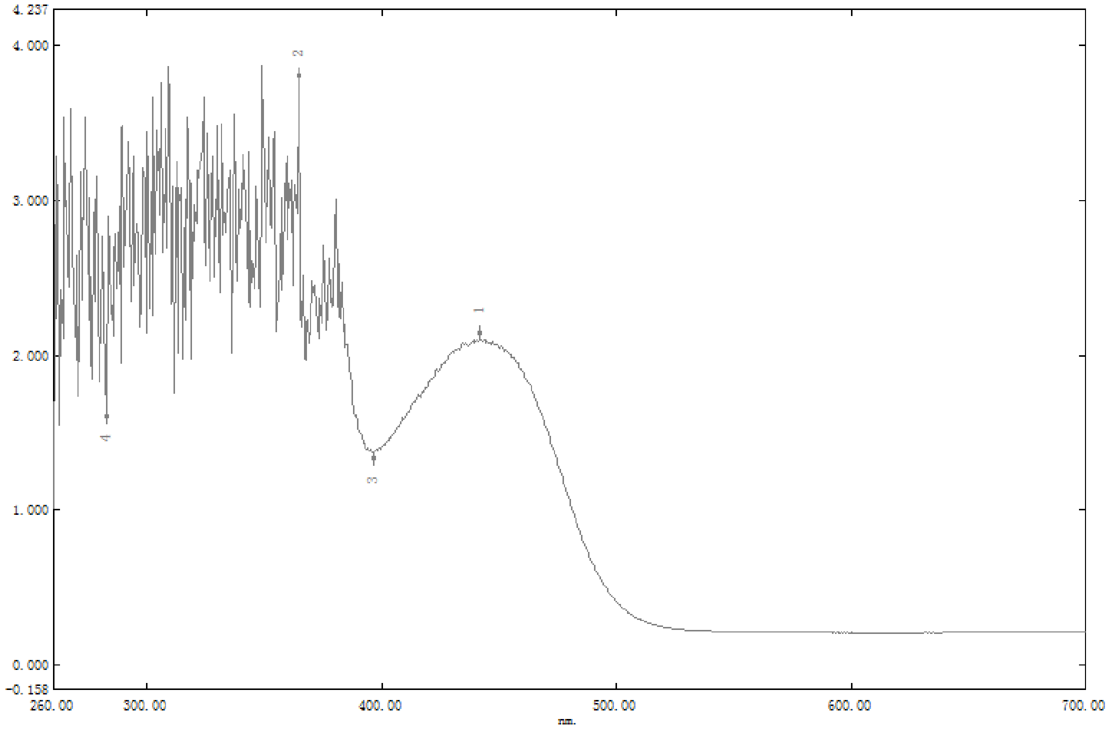

Figure S44 UV-Vis specta of **7h**

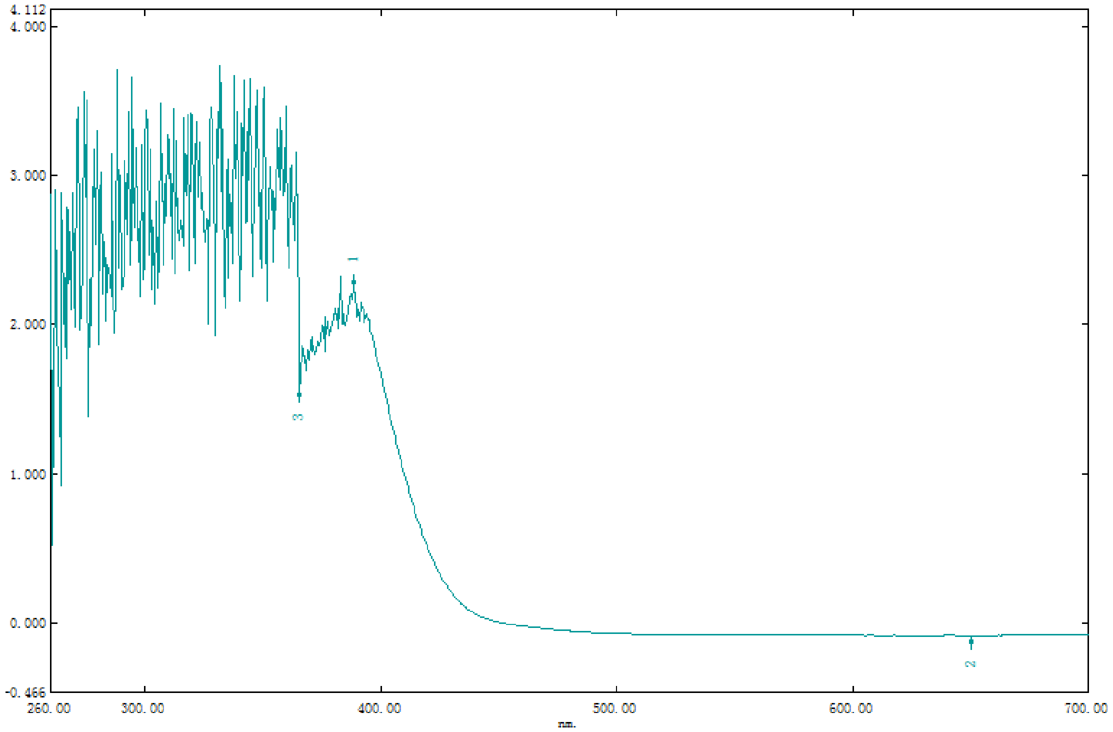

Figure S45 UV-Vis specta of **7i**

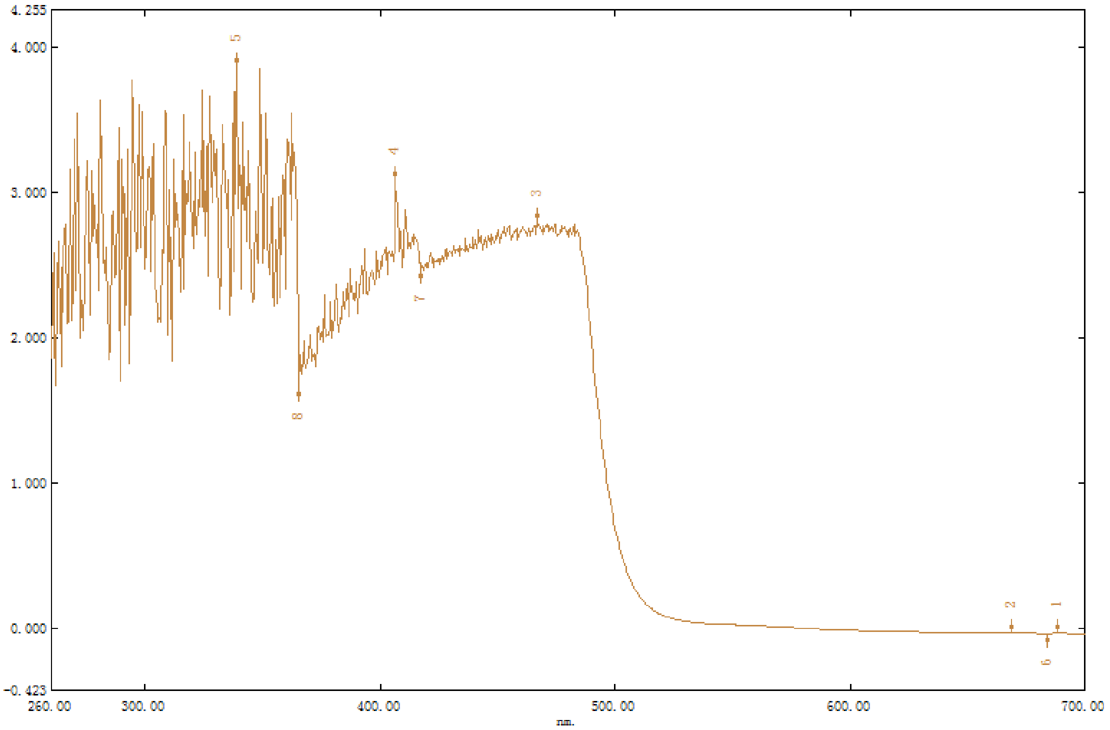

Figure S46 UV-Vis specta of **7j**

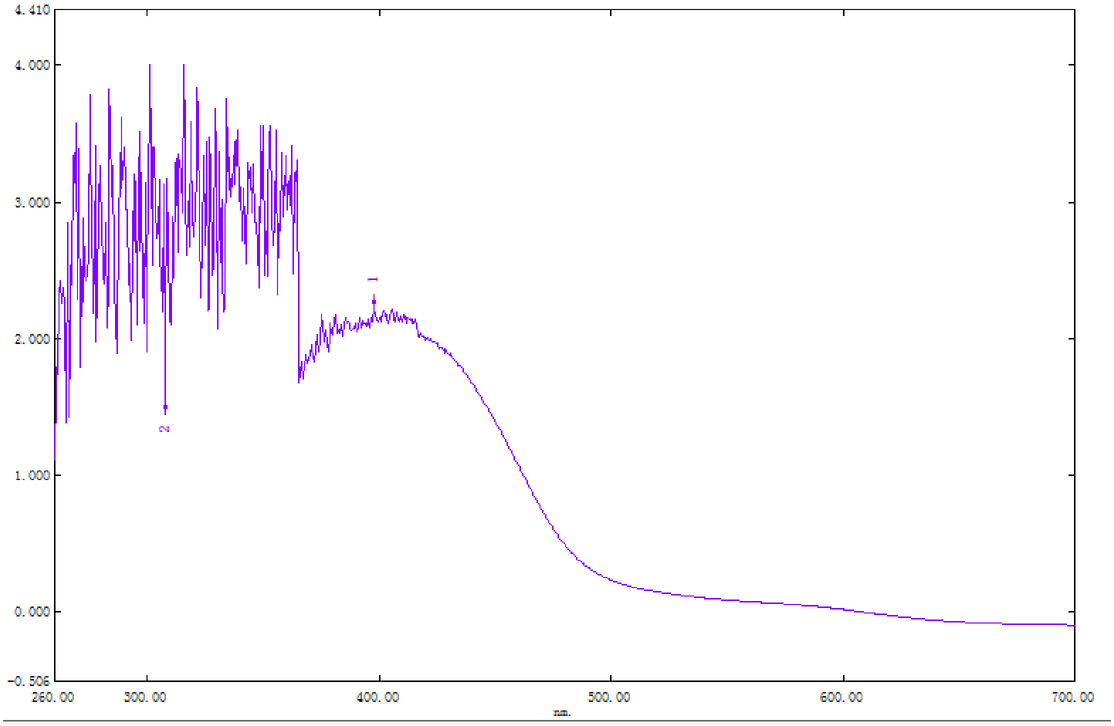

Figure S47 UV-Vis specta of **7k**

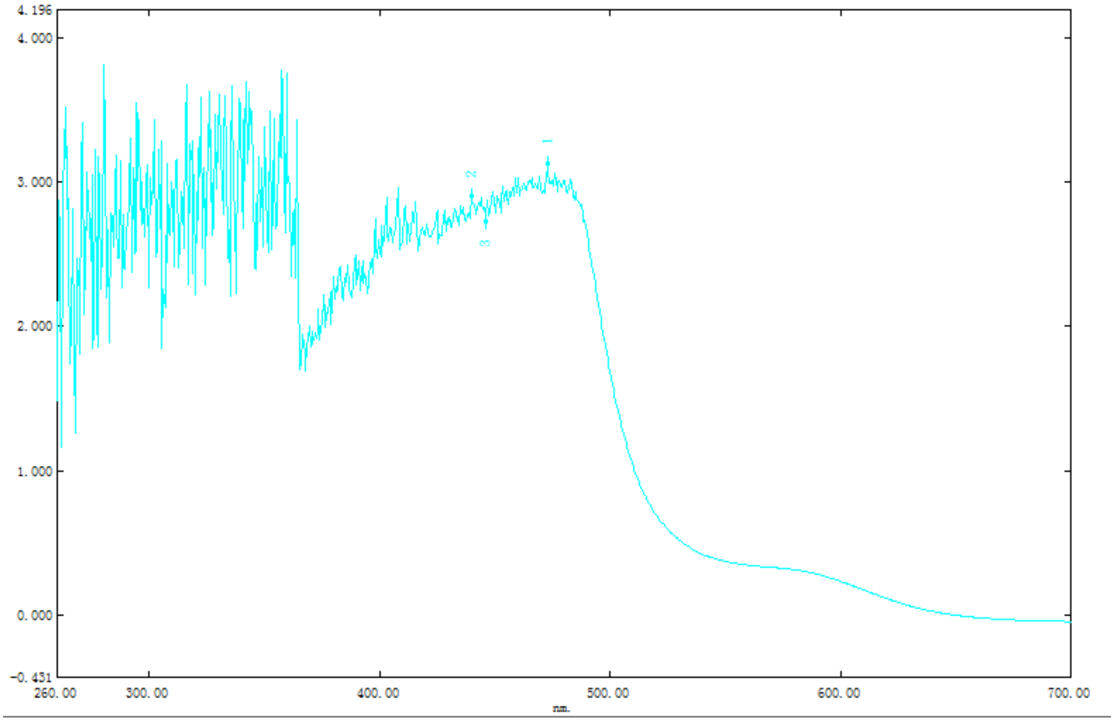

Figure S48 UV-Vis specta of **7l**
